# Co-creating a Data Asset Inventory for Equity-Oriented Research

**DOI:** 10.23889/ijpds.v9i5.2620

**Published:** 2024-09-18

**Authors:** Morgan Stirling, Amy Freier, Nathan Nickel

**Affiliations:** 1 University of Manitoba; 2 Manitoba Centre for Health Policy

**Keywords:** International Migrant, Long-term Resident, Government Assisted and Blended Visa Office-Referred Refugees

## Background

Data asset inventories (DAI) are invaluable resources providing high-level information about datasets, including name, region, purpose, scope, and contents. An effective DAI improves understanding about what data exists, how they can be used, and where they may be limited. Strategies for integrating principles of inclusion, diversity, equity, and accessibility (IDEA) into DAIs are not widely available, which inhibits efforts to improve existing data infrastructure’s capacity for equity-oriented research.

## Approach

Health Data Research Network Canada (HDRN) is a pan-Canadian network that works collaboratively to enable innovative multi-regional research that can improve health and health equity. Central to HDRN’s mandate is strengthening researcher capacity to apply IDEA principles within their research. Within this scope of work, we convened a multi-disciplinary team of computer scientists, data experts, and IDEA specialists to build a DAI of Canadian health and social data using de-stigmatizing language. Through discussion the team identified key data categories and co-created definitions. Data sets were annotated accordingly.

## Results

Discussions highlighted disciplinary differences in understandings of IDEA and its relevance to DAIs. Overcoming these differences required IDEA specialists to be both subject matter experts and advocates. Project delays occurred due to the additional time needed for education. Productive conflict resulted in consensus-building to establish respectful and inclusive terms to describe data.

## Discussion

Embedding IDEA within health data infrastructure requires a lens that may not be associated with one’s training. Developing that lens within a project is possible but requires additional time and effort. These components must be factored into project planning.

